# Network Analysis Identifies *SOD2* mRNA as a Potential Biomarker for Parkinson's Disease

**DOI:** 10.1371/journal.pone.0109042

**Published:** 2014-10-03

**Authors:** Jose A. Santiago, Clemens R. Scherzer, Judith A. Potashkin

**Affiliations:** 1 The Cellular and Molecular Pharmacology Department, The Chicago Medical School, Rosalind Franklin University of Medicine and Science, North Chicago, Illinois, United States of America; 2 The Neurogenomics Laboratory, Harvard Medical School and Brigham and Women's Hospital, Cambridge, Massachusetts, United States of America; Semmelweis University, Hungary

## Abstract

Increasing evidence indicates that Parkinson's disease (PD) and type 2 diabetes (T2DM) share dysregulated molecular networks. We identified 84 genes shared between PD and T2DM from curated disease-gene databases. Nitric oxide biosynthesis, lipid and carbohydrate metabolism, insulin secretion and inflammation were identified as common dysregulated pathways. A network prioritization approach was implemented to rank genes according to their distance to seed genes and their involvement in common biological pathways. Quantitative polymerase chain reaction assays revealed that a highly ranked gene, superoxide dismutase 2 (*SOD2*), is upregulated in PD patients compared to healthy controls in 192 whole blood samples from two independent clinical trials, the Harvard Biomarker Study (HBS) and the Diagnostic and Prognostic Biomarkers in Parkinson's disease (PROBE). The results from this study reinforce the idea that shared molecular networks between PD and T2DM provides an additional source of biologically meaningful biomarkers. Evaluation of this biomarker in *de novo* PD patients and in a larger prospective longitudinal study is warranted.

## Introduction

Accumulating epidemiological evidence suggests a risk of PD among patients with T2DM [1]–[3], reviewed in [4], [5]. However, there remains conflict among some studies. For example, several groups suggest an inverse association between PD and T2DM [6], [7] and other studies have not found a significant association [8], [9]. Despite this discrepancy, T2DM is associated with more severe symptoms in PD. T2DM contributes to postural instability and gait difficulty in PD [10] and insulin resistance is associated with an increased risk of dementia in PD [11]. Besides insulin resistance, dysregulation in other shared biological pathways including mitochondrial dysfunction, endoplasmic reticulum (ER) stress and inflammation may be a plausible explanation for the coexistence of both aging diseases [4], [12].

Both PD and T2DM are considered idiopathic diseases in which a combination of genetic and environmental factors are likely to be involved in the disease pathogenesis. In fact, genetic risk factors identified by genome-wide association studies (GWAS) accounts for approximately 5–10% of the PD and T2DM cases [4], [5]. Several system-biology approaches including animal models and network analysis have been used to understand the molecular mechanisms underlying the linkage between PD and T2DM [5], [13], [14]. For example, diabetic mice treated with 1-methyl-4-phenyl-1,2,3,6-tetrahydropyridine (MPTP) displayed an exacerbated neurodegeneration accompanied by inflammation and ER-stress [13]. In parallel, an integrative network-based approach restricted to data from only GWAS was used to investigate the potential molecular framework linking PD and T2DM and to identify potential biomarkers with clinical applicability. Results from these studies identified the amyloid precursor protein (*APP*) mRNA as a biomarker for PD [15]. Similarly, a network approach identified *PTPN1* mRNA as a diagnostic biomarker in progressive supranuclear palsy, an atypical parkinsonian disorder sometimes misdiagnosed as PD [16].

**Figure 1:**
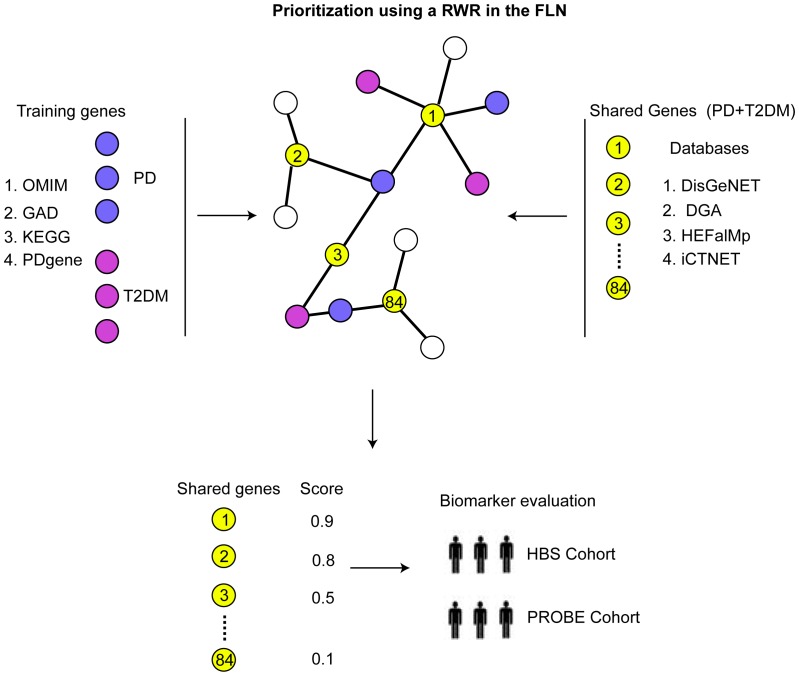
Integrative network approach. Well-characterized genes associated with PD (purple circles) and T2DM (magenta circles) were mapped to the FLN and specified as training set. Shared genes between PD and T2DM (yellow circles) were collected from multiple databases and mapped to the human FLN (black). A random walk algorithm with restart (RWR) was implemented to prioritize the list of shared genes between PD and T2DM according to their distance to known disease genes and in terms of biological pathways involved. A highly ranked gene was evaluated as diagnostic biomarker for PD on RNA samples from whole blood obtained from two independent clinical trials.

Here we expanded our previous network analysis to integrate data from publicly available and curated disease-gene databases to further investigate the connection between both diseases. The disease-gene associations derived from the databases included in this study are not strictly determined by GWAS, thus allowing the exploration of other potentially interesting genes that may have a more modest association. Because PD and T2DM are sporadic and environmental factors play a role in disease etiology and development, it is important to use databases that include genetic disease associations identified through studies that tested environmental factors such as toxin. Our systematic network approach is illustrated in Figure 1. Briefly, genes shared between PD and T2DM were collected from several databases and mapped into the human functional linkage network (FLN). We implemented a random walk algorithm with restart (RWR) to rank the group of genes shared between PD and T2DM. We further evaluated the applicability of the network prioritization approach by testing the most highly ranked gene as a potential diagnostic biomarker for PD. In this study we identify *SOD2* mRNA as a potential blood biomarker that can be used to identify patients with PD.

## Methods

### Database mining and network analysis

We queried the DisGeNET database [17] that integrates information from four respositories: Online Medelian Inheritance in Man (OMIM), UniProt/SwissProte (UNIPROT), Pharmacogenomics Knowledge Base (PHARMGKB), and Comparative Toxicogenomics Database (CTD). DisGeNET can be accessed through the Cytoscape 2.8.3, a platform for complex network analysis [18]. Search disease terms used in DisGeNET were the following: Parkinson Disease (umls:C0030567), Diabetes Mellitus, Type 2 (umls:C0011860). Disease-gene networks were retrieved for PD and T2DM independently. Using the advanced network merge option in Cytoscape, both PD and T2DM gene networks were merged using gene ID as a matching attribute. Only shared genes between both diseases were collected for further analysis.

The Disease and Gene Annotations database (DGA) [19] was accessed through the web (http://dga.nubic.northwestern.edu/pages/search.php). We searched for gene annotations shared between PD and T2DM. Search disease terms in DGA were the following: Parkinson's disease, type 2 diabetes mellitus. Similarly, we explored Human Experimental/Functional Mapper (HEFalMp) using the web-interface (http://hefalmp.princeton.edu/) to investigate genetic associations between PD and T2DM [20]. Search disease terms used in HEFalMp were: Parkinson disease, Diabetes Mellitus. A significance score of 10^−5^ was used as a cut-off value for inclusion in the list of candidate genes. The Integrated Complex Traits Networks interface (iCTNet), can be accessed through the Cytoscape plugin [21]. This database allows the automated construction of disease networks and integrates phenotype-SNP, protein-protein interaction, disease-tissue, tissue-gene and drug-gene interactions. Search disease terms were: Parkinson's disease, Diabetes Mellitus. We queried the disease-gene networks associated with PD and T2DM using a cutoff p-value of 10^-5^. Unlike DisGeNET, disease-gene networks are merged automatically in iCTNet. Like in previous steps, only shared genes between PD and T2DM were collected for further analysis. A total of 84 genes shared between PD and T2DM were collected from the aforementioned databases. Genetic associations were manually curated after searching the literature in Pubmed. Functional and gene ontology analysis was performed using GENEMANIA plugin in Cytoscape [22]. In GENEMANIA, we used the default settings of 20, which are the genes that have the greatest number of interactions, and advanced settings to include physical, predicted, and genetic interactions, and interconnected pathways.

### Gene prioritization methods and cross-validation analysis

The list of 84 genes shared between PD and T2DM collected from the databases was used for subsequent analysis using GPEC, a Cytoscape 2.8.3 plugin that performs a RWR algorithm [23]. We used the default, weighted and undirected human FLN for this analysis that contains 14,230 nodes and 263,884 links. Nodes represent genes and each link represents the likelihood that the connected genes participate in common biological processes. In order to perform the gene prioritization in GPEC, we first collected a list of well-characterized genes associated with PD and T2DM and genes involved in the PD and T2DM KEGG pathways (Table S2). Well-characterized genes known to be associated with PD and T2DM were retrieved from the OMIM (http://www.ncbi.nlm.nih.gov/omim), the Genetic association database (GAD) (http://geneticassociationdb.nih.gov/) and PDgene (http://www.pdgene.org/) (Table S2). Genes involved in the PD and T2DM signaling pathways were retrieved from the KEGG database (http://www.genome.jp/kegg/pathway.html). As a first step in the prioritization, the list of well-characterized genes associated with PD and the PD KEGG pathway was used as a training set. The test set included the 84 genes shared between both diseases and genes associated with T2DM and its associated KEGG pathway. The training set was manually curated to ensure that there was no overlap with any of the genes contained in the test set. To perform the RWR, we set back-probability to 0.5 and candidate genes were scored and ranked. As a second step, we performed a series of prioritization steps with respect to the most significant biological pathways retrieved by GENEMANIA. These prioritization steps were performed for each individual pathway independently. To this end, we collected the set of genes curated for each biological pathway from the Broad Institute's Molecular Signatures Database (MSigDB) 3.0 [24] (Table S2). Here, the training set consisted of genes curated for each pathway and the test set consisted of the 84 genes shared between PD and T2DM. In GPEC, we evaluated the performance of each prioritization with a leave-one-out cross-validation (LOOCV) strategy where the number of training genes is equal to the number of cross-validation trials and one of the genes in the test set is held out during each trial. As a result, a ROC curve of sensitivity versus 1-specificity is built by the software. Since all the scores were determined by the RWR algorithm, the final score for each gene was defined as the sum of all individual scores obtained from each prioritization as previously demonstrated using similar analyses [15], [16], [25]. The overall workflow is presented in Figure 1.

### Information about HBS and PROBE study participants

The Institutional Review Boards of Rosalind Franklin University of Medicine and Science approved the study protocol. Written informed consent was received from all participants. We used 96 individuals including 50 PD patients (31 men, 19 women; Hoehn and Yahr scale 1.97±0.62; mean age at enrollment 63.12±8.96; mean age at onset 58.75±10.17) and 46 healthy age-matched controls (HC) (26 men, 20 women; mean age at enrollment 64.28±10.42) enrolled in the HBS. Other clinical information is reported in [15]. There were 5 PD and 5 HC patients with T2DM. Details of patient and controls recruitment, clinical assessments, and biobanking in the HBS study population have been reported in part elsewhere [26] and http://www.neurodiscovery.harvard.edu/research/biomarkers.html. As an independent replication set, we used 51 PD patients (29 men, 22 women; mean age at enrollment 63.16±6.42; Hoehn and Yahr scale 2±0.28) and 45 HC (24 male, 21 women; mean age at enrollment 65.12±8.60) enrolled in the PROBE Study (#NCT00653783). There was one HC patient with T2DM. Clinical diagnosis of PD was based on the United Kingdom Parkinson's Disease Society Brain Bank criteria. Healthy controls had no history of neurological disease and a Mini-Mental State Examination (MMSE) test score higher than 27. Inclusion and exclusion criteria for patients enrolled in the PROBE study are reported elsewhere in [27].

### RNA isolation and real time polymerase chain reactions

Blood was collected and prepared as described using the PAXgene Blood RNA system (Qiagen,Valencia, CA, USA)[28]. Samples with RNA integrity values > 7.0 and ratio of absorbances at 260/280 nm between 1.7 and 2.4 were used in the current study. Primer Express software (Life Technologies, Carlsbad, CA, USA) was used to design the primers. The High Capacity RNA transcription kit (Life Technologies, Carlsbad, CA, USA) was used to reverse transcribe 1 µg of total RNA according to the manufacturer's protocol. The DNA engine Opticon 2 Analyzer (Bio-Rad Life Sciences, Hercules, CA, USA) was used for the qPCR reactions. Each 25 µl reaction contained Power SYBR (Life Technologies, Carlsbad, CA, USA) and primers at a concentration of 5 µM. Primer sequences used in qPCR assays are as follows: *GAPDH*; forward: 5′- CAACGGATTTGGTCGTATTGG-3′; reverse: 5′- TGATGGCAACAATATCCACTTTACC-3′, *SOD2*; forward: 5′- GTTCAATGGTGGTGGTCATATCA-3′; reverse: 5′- GCAACTCCCCTTTGGGTTCT-3′. Amplification conditions and detailed description of qPCR experiments is described in [15].

### Statistical analysis

All analyses were performed with Prism4.0 (GraphPad, La Jolla, CA, USA) and Statistica 8.0 (Statsoft, OK, Tulsa, USA). A student t-test (two-tailed) was used to estimate the significance between PD cases and controls for numerical variables. Linear regression and Pearson correlation analysis was used to determine statistical significance for the prospective biomarker adjusting for sex, age, Hoehn & Yahr scale in both cohorts of patients and body mass index (BMI) in the HBS study. A ROC curve analysis was used to evaluate the diagnostic accuracy. A p-value less than 0.05 was regarded statistically significant.

## Results

### Identification of shared genes between PD and T2DM from disease-gene databases

We explored the DisGeNET database, a comprehensive database of the human genetic associations related to disease [17]. In DISGENET, the central node represents the disease and the nodes linked to the central node represent genes that have been associated to the queried disease. We queried the disease-gene networks associated with both PD and T2DM. Analysis of the merged network revealed a cluster consisting of 53 shared genes between PD and T2DM (Table S1, Methods).

We next explored the DGA interface [19] and found 42 overlapping genes with the gene set collected in DisGeNET and 8 additional genes shared between PD and T2DM (Table S1). We next interrogated the HEFalMp interface [20]. Similarly to DGA and DisGeNET, we collected the shared genes between PD and T2DM. The most significant genes in T2DM associated to PD were *HNF4A*, *PDX1*, *SLC2A4*, and *ABCC8* (Q<10^−05^)(Table S1). Finally, we interrogated the iCTNet interface [21] that contains results from 118 GWAS published studies and data from the GWAS catalog. In iCTNet, we found 20 genes shared between both diseases (Table S1). A total of 84 genes shared between PD and T2DM were collected from the aforementioned databases and used for further analysis.

**Figure 2:**
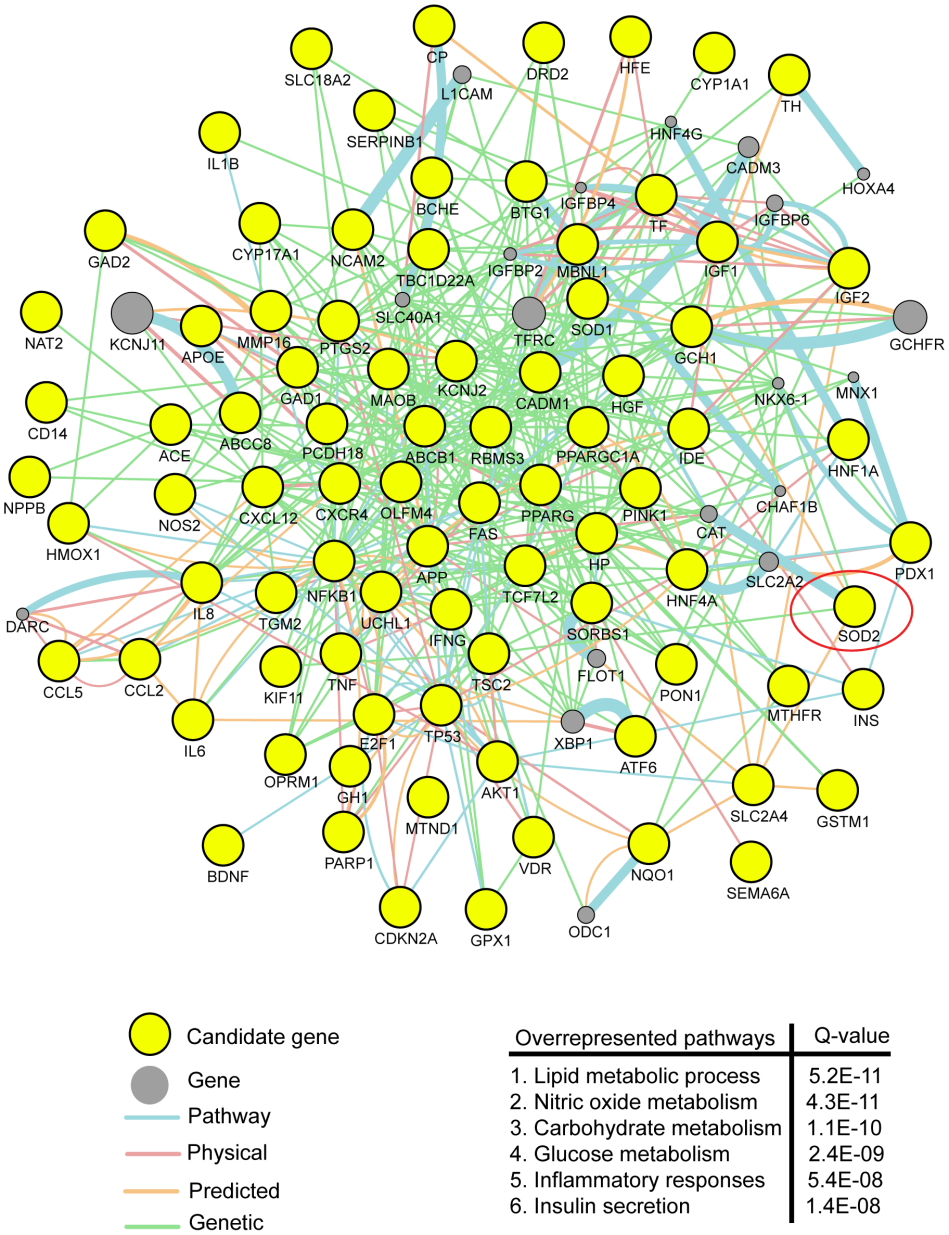
Biological functional analysis of candidate genes. Network of interactions among PD and T2DM shared genes, as retrieved by GeneMANIA. Shared genes between PD and T2DM are displayed in yellow circles and other genes with the greatest number of interactions with shared genes are displayed in gray circles. The size of the gray nodes represents the degree of association with the input genes (i.e., smaller size represents low connectivity). The most represented pathways retrieved by GeneMANIA are displayed using GO annotations and Q-values of significance.

To further identify the potential functional implications in the cluster of genes shared between PD and T2DM, we imported all 84 genes into GeneMANIA [22]. Analysis of the 84 shared genes identified the most overrepresented pathways including nitric oxide biosynthetic processing, carbohydrate and lipid metabolic processing, insulin secretion, regulation of glucose, and inflammation (Figure 2).

### Gene prioritization and experimental validation

Given the numerous molecular links between PD and T2DM, we investigated the extent to which genes identified as shared between PD and T2DM can be used to classify patients with PD. This idea is particularly salient in light of the recent finding that revealed that genes identified in shared molecular networks between PD and T2DM may improve the clinical diagnosis of PD. Accordingly, APP was identified in a functional network shared between well-characterized genes associated with PD and T2DM. APP mRNA was capable to distinguish PD patients from HC with 80% accuracy [15], a diagnostic capacity that extends beyond the one afforded by the current clinical diagnostic criteria [29], [30].

We implemented a candidate prioritization approach using a RWR algorithm within the human the FLN described previously [15], [16], [25], [31]. This algorithm measures the closeness of potentially candidate genes to confirmed genes within the FLN or protein-protein interaction network. We used GPEC, a cytoscape plugin for RWR-based gene prioritization [23] to rank 84 candidates collected from the curated databases (Table S1). In the RWR algorithm, the known disease genes are mapped to the FLN and specified as “training set” and the “test set” containing potential candidates can be ranked according to their closeness to the training genes within the FLN (See Methods). The training set consisted of well-characterized genes associated with PD and its KEGG associated pathway. The test set included the list of 84 shared genes and well-characterized genes associated with T2DM and its KEGG associated pathway. RWR score-based genes are listed in Table S3. Further, we evaluated the performance of the gene prioritization using a LOOCV strategy (see Methods). LOOCV represented in terms of receiver operating characteristic curve (ROC) resulted in an area under curve AUC_PD-T2DM_ value of 0.85 (Figure S1A).

As a second step, we prioritized the list of 84 shared genes with respect to the most significant biological pathways determined by GeneMania (see Methods). We collected the set of genes curated for each biological pathway from the Broad Institute's Molecular Signatures Database 3.0 (MSigDB) [24] (Table S2). These gene sets were used as training sets during each prioritization. Gene prioritization was performed in GPEC for each individual pathway independently (Methods, Table S3). LOOCV performed for each prioritization resulted in AUC values ranging from 0.90–0.99 (Figure S1B-E). The top RWR score-based genes are listed in Table 1. The complete list of RWR score-based candidate genes according to each prioritization step is provided in (Table S3).

**Table 1:** Highly ranked RWR score-based genes.

Rank	Gene Symbol	Gene Name	Score
1	*SOD2*	Superoxide dismutase 2	3.08E-03
2	*MT-ND1*	Mitochondrially encoded NADH dehydrogenase 1	2.93E-03
3	*IFNG*	Interferon, gamma	2.90E-03
4	*TNF*	Tumor necrosis factor	2.39E-03
5	*TP53*	Tumor protein p53	2.36E-03
6	*IL6*	Interleukin 6	2.16E-03
7	*AKT1*	V-akt murine thymoma viral oncogene homolog 1	1.96E-03
8	*HNF4A*	Hepatocyte nuclear factor 4, alpha	1.80E-03
9	*HMOX1*	Heme oxygenase (decycling) 1	1.77E-03
10	*FAS*	Fas (TNF receptor superfamily, member 6)	1.53E-03
11	*APP*	Amyloid beta (A4) precursor protein	1.34E-03
12	*CYP17A1*	Cytochrome P450, family 17, subfamily A, polypeptide 1	1.23E-03
13	*IGF1*	Insulin-like growth factor 1	1.03E-03
14	*PTGS2*	Prostaglandin-endoperoxide synthase 2	1.02E-03
15	*SOD1*	Superoxide dismutase 1, soluble	9.80E-04
16	*BDNF*	Brain-derived neurotrophic factor	8.46E-04
17	*NOS2*	Nitric oxide synthase 2	8.34E-04
18	*TGM2*	Transglutaminase 2	6.86E-04
19	*GCH1*	GTP cyclohydrolase 1	6.66E-04
20	*UCHL1*	Ubiquitin carboxyl-terminal esterase L1	6.60E-04

**Figure 3:**
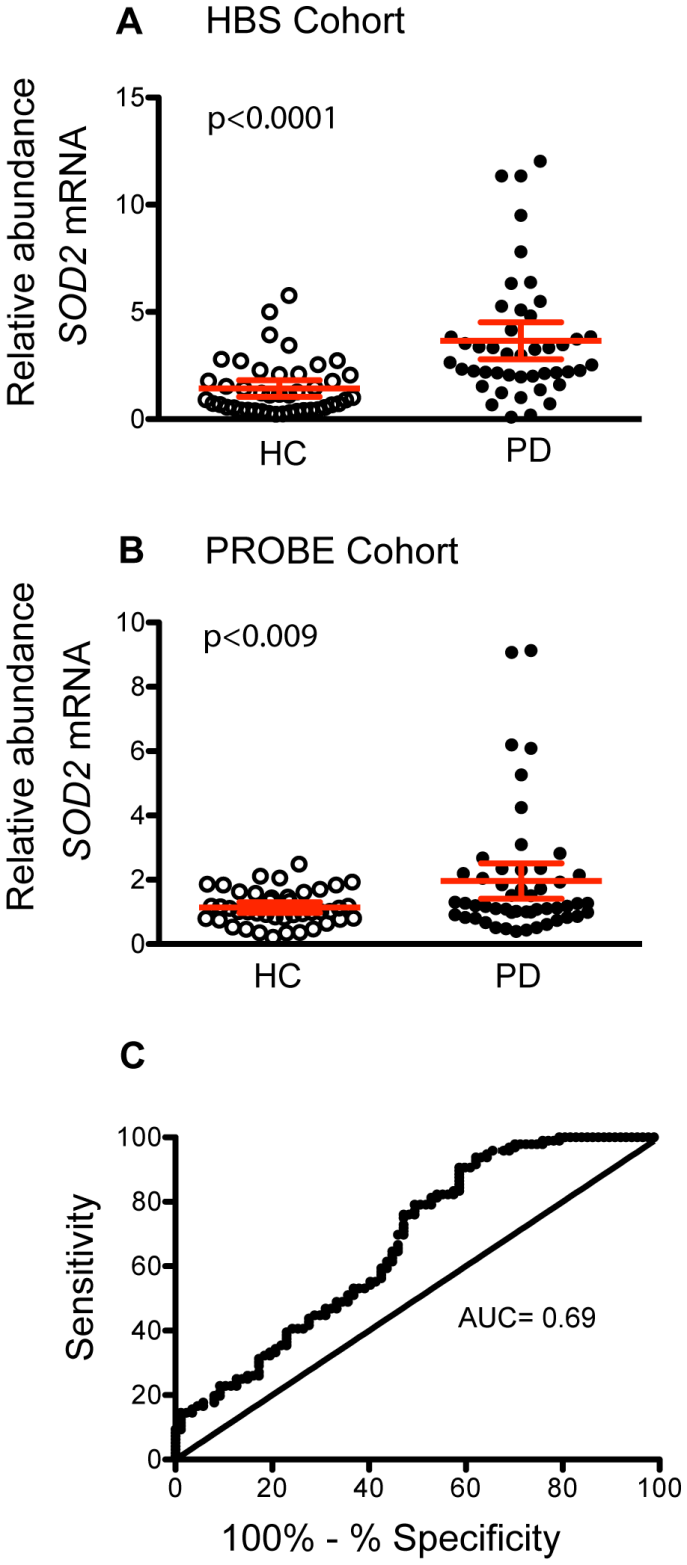
Evaluation of *SOD2* as a potential biomarker for PD. **A.** Relative abundance of *SOD2* mRNA in blood of PD patients (black circles) compared to healthy controls (white circles) in samples from the HBS cohort. **B.** Replication of biomarker expression in an independent set of samples from patients enrolled in the PROBE study. Relative abundance of each biomarker was calculated using *GAPDH* as a reference gene and healthy controls as calibrator. Error bars represent standard error. **C.** ROC curve analysis to evaluate the diagnostic accuracy of *SOD2*.

In order to validate the results obtained from the network analysis we evaluated the most highly ranked gene, *SOD2*, as a potential biomarker for PD. Relative abundance of *SOD2* mRNA was measured in whole blood of PD patients compared to HC from samples obtained from two independent clinical trials, HBS and PROBE. Quantitative PCR assays revealed that *SOD2* mRNA is significantly upregulated in blood of PD patients compared to HC in both cohorts of study participants, although significant overlap in expression levels was observed between PD and controls (Figure 3A and B). To evaluate the diagnostic accuracy of *SOD2* in distinguishing PD patients from HC, ROC curve analysis was performed. As shown in Figure 3C, the AUC values for *SOD2* was 0.69.

Pearson correlation analysis demonstrated that relative abundance of *SOD2* was independent of other covariates including age (r = −0.13, p = 0.40), and sex (r = −0.03, p = 0.79) in both cohorts of patients and BMI (r = 0.18, p = 0.21) in the HBS cohort. Correlation analysis of *SOD2* mRNA expression and Hoehn and Yahr stage was not significant (r = 0.04, p = 0.73). Correlation with medication was not determined since most of the patients with PD were medicated with several drugs and the number of untreated patients was too small to reliably detect a significant change.

## Discussion

We have recently demonstrated that shared molecular networks between PD and T2DM can be exploited to identify highly accurate biomarkers for PD [15]. This result along with other studies investigating the relationship between PD and T2DM suggested that a system-level understanding of the comorbidity between both diseases might improve the clinical management of PD and may elucidate potential therapeutic targets [5], [13].

In our previous study the network analysis was restricted to the intersection of genes between PD and T2DM identified by GWAS. However, this approach is limited given that a large number of genes fail to exceed the genome-wide statistical threshold of significance and are therefore neglected. One example was the failure to recognize the association of a polymorphism in PPARG in T2DM by other studies because of its modest effect on susceptibility for T2DM [34]. In addition, very few causative genes of disease have been proven to be useful for clinical diagnosis [35]. For example, mutations in *LRRK2* and *DJ-1* are implicated in hereditary PD, but changes in their mRNA or protein expression levels in blood may not be useful diagnostic biomarkers for early stage PD [36], [37]. Here we expanded our analysis to integrate data from publicly available databases that includes a wide range of experimental designs including but not limited to pharmacogenomics, toxicogenomics, and other experiments in addition to GWAS.

We identified 84 genes shared between PD and T2DM by interrogating several disease-gene databases. Biological and functional analysis of these genes identified shared dysregulated pathways including nitric oxide biosynthesis, regulation of glucose, lipid and carbohydrate metabolism, insulin secretion and inflammation. Shared genes between both diseases were prioritized using a RWR within the human FLN. Not surprisingly, highly ranked genes were representative of the most significant dysregulated pathways. For example, *AKT1*, *IGF1* and *TP53* are involved in insulin signaling and glucose homeostasis [38], [39]. In this regard, dysregulation of glucose metabolism was identified as an early event in sporadic PD and it has been hypothesized that alpha synuclein (SNCA) may play a role in this process [40]. In addition, genes associated with inflammation including *TNF* and *IL6* are among the most highly ranked genes. In this context, neuroinflammation is associated with the pathophysiology of PD [41], [42]. *HNF4A* was also among the top 10 genes in the prioritized list. Interestingly, *HNF4A* may also play a role in intestinal lipid metabolism, oxidative stress and inflammation, processes that are implicated in both chronic diseases [43]. Collectively, these findings are consistent with previous studies that highlight the significant convergence of dysregulated pathways in PD and T2DM [4], [15], [44].

We further evaluated a highly ranked gene, *SOD2* in blood of patients with PD from two independent cohorts of study participants. Relative abundance of SOD2 was upregulated in blood of PD patients compared to healthy individuals. *SOD2* is a mitochondrial enzyme that protects against oxidative stress by converting superoxide radicals to molecular oxygen and hydrogen peroxide. Given its antioxidant capacity, it has been implicated in the pathogenesis of PD. For example, inactivation of SOD2 increases mitochondrial ROS production in *in vitro* models of PD [45]. Moreover, SOD2 protein levels are increased in the frontal cortex of PD patients [46]. In the context of diabetes, increased levels of *SOD2* mRNA have been found in skeletal muscle of patients with T2DM [47]. In addition, SOD2 has been associated to be involved in inflammation [48], insulin signaling and glucose metabolism[49], [50], and lipid metabolism and peroxidation [51], processes that were identified dysregulated in the network analysis. Therefore, it is not surprising that SOD2 was the most highly ranked gene by the prioritization method.

Recently, drugs to treat diabetic patients, metformin-sulfonylurea and exenatide have shown promise in PD patients [52], [53]. In fact, improvement of motor and cognitive functions persists one year after the treatment with exenatide [54]. Interestingly, diabetic drugs are known to interact with SOD2. For example, metformin treatment results in an increased expression of *SOD2* mRNA in human endothelial cells [55]. Troglitazone treatment, another anti-diabetic and anti-inflammatory drug, results in decreased expression of *SOD2* mRNAs in cellular models [56], [57]. In addition, gliclazide treatment, an oral sulfonylurea hypoglycemic agent, results in decreased protein expression of SOD2 [58], and rosiglitazone, an insulin sensitizer, increased SOD2 protein expression in retinal cells from mice [59]. Based on these observations, expression of *SOD2* in blood may be useful to evaluate the therapeutic effect of anti-diabetic drugs in PD patients.

This study has several strengths and limitations. Biomarkers obtained from microarray studies may be data set specific and not indicative of the underlying disease pathology. In this context, our integrated network approach provides a framework to identify and prioritize PD biomarkers involved in common dysregulated pathways. Another strength is the replication of this biomarker in two independent cohorts of patients. However, there are several limitations and potential confounding factors. For example, although we have found that *GAPDH* mRNA expression in blood is stable in previous studies [15], [16], [27], [60], replication of this biomarker using several reference genes for normalization is desirable [61]. In addition, differences in blood counts and PD medications may bias gene expression results. Thus, evaluation of *SOD2* mRNA in drug-naïve PD patients and in a large well-characterized prospective study will be important to determine its clinical utility.

In summary, our study demonstrates that integration of shared molecular networks provides a useful framework to prioritize candidate biomarkers in a biologically relevant context. Remarkably, we demonstrate that expression of a highly ranked gene identified within shared dysregulated pathways can be used as diagnostic marker for PD. We foresee integrated network approaches will provide a better understanding of the underlying disease mechanism and facilitate the discovery of accurate biomarkers and therapeutic targets. In this regard, a network-based approach was useful to identify a neuroprotective agent, alvespimycin (17-DMAG), in PD [25]. Although the prioritization method presented in this study has been evaluated in the specific case of PD-T2DM, other disease-disease associations may be studied following this protocol. For instance, the construction of shared genes and protein networks have facilitated the understanding of other disease-disease associations such as asthma and tuberculosis [32] and artherosclerosis-induced ocular diseases [33]. Thus, network analysis of disease comorbidities may reveal novel diagnostic biomarkers and therapeutic strategies.

Future studies will be aimed to replicate these findings in samples from non-medicated and patients at risk of PD and to evaluate other potential candidate biomarkers found in this study.

## Supporting Information

Figure S1 **Validation of each prioritization step.** The performance of each prioritization step was validated by computing values for ROC and AUC through the leave-one-out validation method using GPEC.
(TIF)

Table S1 **PD and T2DM shared cluster of genes. 84 shared genes between PD and T2DM and the corresponding databases from which each gene was collected.**
(DOC)

Table S2 **Curated gene sets used for RWR prioritization.**
(DOC)

Table S3 **RWR-based scores for each prioritization within the functional linkage network.** Score PD-T2DM is the score for the disease prioritization, p1 is insulin signaling pathway, p2 is nitric oxide biosynthesis, p3 is glucose metabolism, p4 is inflammation, p5 is lipid metabolism and c is the cumulative score.
(DOC)
