# Supplementary figures and images for: Network Analysis Identifies SOD2 mRNA as a Potential Biomarker for Parkinson's Disease
Source: PLoS One. 2014 Oct 3;9(10):e109042. doi: 10.1371/journal.pone.0109042 (PMC4184821; doi:10.1371/journal.pone.0109042)

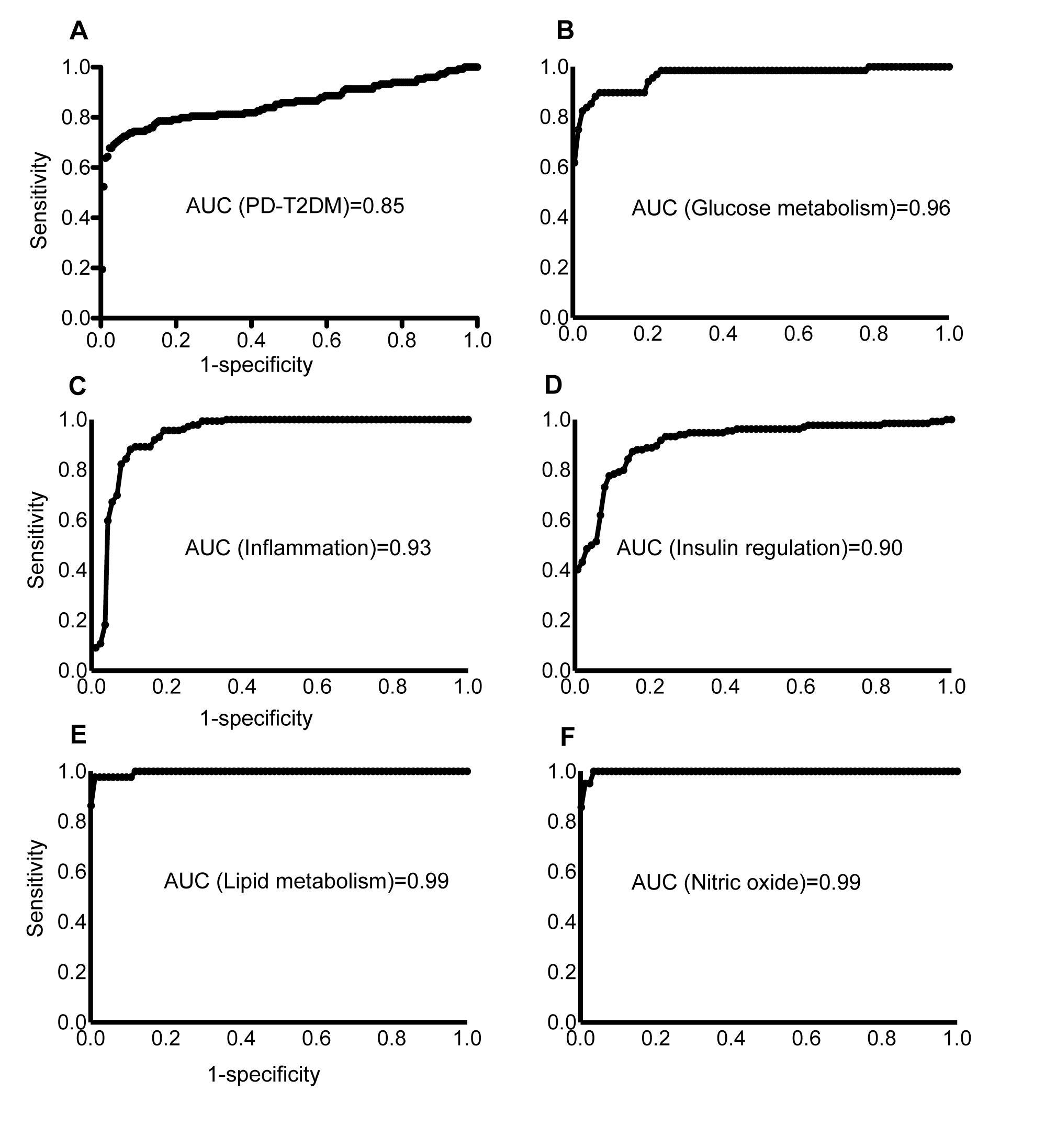

Supplement: Figure S1 — Validation of each prioritization step. The performance of each prioritization step was validated by computing values for ROC and AUC through the leave-one-out validation method using GPEC. (TIF) [file pone.0109042.s001.tif]
