# Supplementary material for: Network Analysis Identifies SOD2 mRNA as a Potential Biomarker for Parkinson's Disease
Source: PLoS One. 2014 Oct 3;9(10):e109042. doi: 10.1371/journal.pone.0109042 (PMC4184821; doi:10.1371/journal.pone.0109042)
Supplement: Table S1 — PD and T2DM shared cluster of genes. 84 shared genes between PD and T2DM and the corresponding databases from which each gene was collected. (DOC) [file pone.0109042.s002.doc]

**Supplementary Table 1.** PD and T2DM shared cluster of genes.

| **Gene Symbol** | **Entrez ID** | **Database** |
| --- | --- | --- |
| *VDR* | 7421 | DisGeNET, DGA |
| *TNF* | 7124 | DisGeNET, DGA |
| *TH* | 7054 | DisGeNET, DGA |
| *TF* | 7018 | DisGeNET, DGA |
| *SOD2* | 6648 | DisGeNET, DGA |
| *PTGS2* | 5743 | DisGeNET, DGA |
| *PON1* | 5444 | DisGeNET, DGA |
| *PINK1* | 65018 | DisGeNET, DGA |
| *PARP1* | 142 | DisGeNET, DGA |
| *NQO1* | 1728 | DisGeNET, DGA |
| *MTND1* | 4535 | DisGeNET, DGA |
| *NAT2* | 10 | DisGeNET, DGA |
| *MTHFR* | 4524 | DisGeNET, DGA |
| *INS* | 3630 | DisGeNET, DGA |
| *IL1B* | 3553 | DisGeNET, DGA |
| *IFNG* | 3458 | DisGeNET, DGA |
| *IL1B* | 3553 | DisGeNET, DGA |
| *HP* | 3240 | DisGeNET, DGA |
| *HMOX1* | 3162 | DisGeNET, DGA |
| *HFE* | 3077 | DisGeNET, DGA |
| *GPX1* | 2876 | DisGeNET, DGA |
| *FAS* | 355 | DisGeNET, DGA |
| *DRD2* | 1813 | DisGeNET, DGA |
| *CYP1A1* | 1543 | DisGeNET, DGA |
| *CYP17A1* | 1586 | DisGeNET, DGA |
| *CDKN2A* | 1029 | DisGeNET, DGA |
| *CD14* | 929 | DisGeNET, DGA |
| *CCL5* | 6352 | DisGeNET, DGA |
| *CCL2* | 6347 | DisGeNET, DGA |
| *APOE* | 348 | DisGeNET, DGA |
| *ACE* | 1636 | DisGeNET, DGA |
| *ABCB1* | 5243 | DisGeNET, DGA |
| *AKT1* | 207 | DisGeNET, DGA |
| *CP* | 1356 | DisGeNET, DGA |
| *GCH1* | 2643 | DisGeNET, DGA |
| *GSTM1* | 2944 | DisGeNET, DGA |
| *IGF1* | 3479 | DisGeNET, DGA |
| *IL8* | 3576 | DisGeNET, DGA |
| *MAOB* | 4129 | DisGeNET, DGA |
| *SLC18A2* | 6571 | DisGeNET, DGA |
| *SOD1* | 6647 | DisGeNET, DGA |
| *UCHL1* | 7345 | DisGeNET, DGA |
| *OPRM1* | 4988 | DisGeNET |
| *NPPB* | 4879 | DisGeNET |
| *NOS2* | 4843 | DisGeNET |
| *NFKB1* | 4790 | DisGeNET |
| *IL6* | 3569 | DisGeNET |
| *IDE* | 3416 | DisGeNET |
| *HGF* | 3082 | DisGeNET |
| *BDNF* | 627 | DisGeNET |
| *ATF6* | 22926 | DisGeNET |
| *ADH1C* | 126 | DisGeNET |
| *APP* | 351 | DGA |
| *E2F1* | 1869 | DGA |
| *GAD1* | 2571 | DGA |
| *GAD2* | 2572 | DGA |
| *GH1* | 2688 | DGA |
| *TGM2* | 7052 | DGA |
| *TP53* | 7157 | DGA |
| *TSC2* | 7249 | DGA |
| *BTG1* | 694 | iCTNet |
| *HNF4A* | 3172 | iCTNet, HEFalMp |
| *SEMA6A* | 57556 | iCTNet |
| *CXCR4* | 7852 | iCTNet |
| *RBMS3* | 27303 | iCTNet |
| *PPARG* | 5468 | iCTNet, DGA |
| *HNF1A* | 6927 | iCTNet |
| *TCF7L2* | 6934 | iCTNet |
| *TBC1D22A* | 25771 | iCTNet, DisGeNET |
| *OLFM4* | 10562 | iCTNet |
| *SORBS1* | 10580 | iCTNet |
| *CADM1* | 23705 | iCTNet |
| *PCDH18* | 54510 | iCTNet |
| *NCAM2* | 4685 | iCTNet |
| *MMP16* | 4325 | iCTNet |
| *SERPINB1* | 1992 | iCTNet |
| *PPARGC1A* | 10891 | iCTNet, DGA |
| *MBNL1* | 4154 | iCTNet |
| *KIF11* | 3832 | iCTNet |
| *KCNJ2* | 3759 | iCTNet |
| *CXCL12* | 6387 | iCTNet |
| *PDX1* | 3651 | HEFalMp |
| *SLC2A4* | 6517 | HEFalMp |
| *ABCC8* | 6833 | HEFalMp |
