# Supplementary material for: Network Analysis Identifies SOD2 mRNA as a Potential Biomarker for Parkinson's Disease
Source: PLoS One. 2014 Oct 3;9(10):e109042. doi: 10.1371/journal.pone.0109042 (PMC4184821; doi:10.1371/journal.pone.0109042)
Supplement: Table S2 — Curated gene sets used for RWR prioritization. (DOC) [file pone.0109042.s003.doc]

Supplementary Table 2. Curated gene sets used for RWR prioritization.

| **Disease or biological pathway** | **Gene sets** |
| --- | --- |
| Parkinson’s disease | KEGG 05012: PD signaling pathway  PDgene, GAD, OMIM: *GAK, DGKQ, STH, MAPT, LRRK2, SNCA, LOC642072, WNT3, RIT2, GBA, MCCC1, LAMP3, SCARB2, SYT11, ACMSD, STK39, BST1, HLA-DRB5, CCDC62, HIP1R, HLA-DRA, PARK16, SLC45A3, NUCKS1, RAB7L1, SLC41A1, PM20D1, C17ORF69, KIAA1267, LOC644246, NSF, FAM47E, SREBF1, TMEM175, BRDG1, DLG2, PLEKHM1, IMP5, CRHR1, PM20D1* |
| Type 2 diabetes | KEGG 04930: T2DM signaling pathway  GAD and OMIM: *ARF5, PAX4, SND1, IGF2BP2,*  *GRK5, RASGRP1, GLIS3, CDKN2B, CDC123, HNF1B, FAM58A, DUSP9, CDKAL1, LAMA1, FTO, HHEX, RBM43, RND3, MAEA, GLIS3, FITM2, R3HDML, GCC1, PSMD6, ZFAND3, HMG20A, AP3S2, KCNQ1, SPRY2, C2CD4A, C2CD4B, BCL11A, ZBED3, KLF14, TP53INP1, CENTD2, HMGA2, ZFAND6, PRC1, IRS1, MTNR1B, JAZF1, IDE, SRR, PTPRD, SLC30A8, CAMK1D, TSPAN8, LGR5, THADA* |
| Insulin signaling | KEGG: 04910 |
| Nitric oxide biosynthesis | MSigDB: M11650 BioCarta: Nitric oxide signaling pathway |
| Glucose metabolism | MSigDB: M1879 Reactome glucose metabolism |
| Inflammation | KEGG: 04062 |
| Lipid metabolism | KEGG: 00071 |
