# Supplementary material for: Network Analysis Identifies SOD2 mRNA as a Potential Biomarker for Parkinson's Disease
Source: PLoS One. 2014 Oct 3;9(10):e109042. doi: 10.1371/journal.pone.0109042 (PMC4184821; doi:10.1371/journal.pone.0109042)
Supplement: Table S3 — RWR-based scores for each prioritization within the functional linkage network. Score PD-T2DM is the score for the disease prioritization, p1 is insulin signaling pathway, p2 is nitric oxide biosynthesis, p3 is glucose metabolism, p4 is inflammation, p5 is lipid metabolism and c is the cumulative score. (DOC) [file pone.0109042.s004.doc]

**Supplementary Table 3**. RWR-based scores for each prioritization within the functional linkage network. Score PD-T2DM is the score for the disease prioritization, p1 is insulin signaling pathway, p2 is nitric oxide biosynthesis, p3 is glucose metabolism, p4 is inflammation, p5 is lipid metabolism and c is the cumulative score.

| **Rank** | **Gene** | **Score (PD-T2DM)** | **Score (p1)** | **Score (p2)** | **Score (p3)** | **Score (p4)** | **Score (p5)** | **Score (c)** |
| --- | --- | --- | --- | --- | --- | --- | --- | --- |
| 1 | *SOD2* | 9.07E-04 | 1.49E-04 | 2.21E-04 | 1.37E-03 | 3.73E-05 | 3.96E-04 | 3.08E-03 |
| 2 | *MTND1* | 2.68E-03 | 1.41E-05 | 1.16E-05 | 1.47E-04 | 1.94E-06 | 7.71E-05 | 2.93E-03 |
| 3 | *TNF* | 2.18E-04 | 4.75E-04 | 1.38E-04 | 2.46E-04 | 1.76E-03 | 5.96E-05 | 2.90E-03 |
| 4 | *IFNG* | 7.27E-05 | 2.99E-04 | 7.12E-05 | 5.00E-05 | 1.88E-03 | 2.39E-05 | 2.39E-03 |
| 5 | *TP53* | 5.18E-04 | 5.58E-04 | 2.70E-04 | 3.90E-04 | 3.96E-04 | 2.26E-04 | 2.36E-03 |
| 6 | *IL6* | 7.38E-05 | 3.67E-04 | 1.01E-04 | 5.93E-05 | 1.53E-03 | 3.17E-05 | 2.16E-03 |
| 7 | *AKT1* | 2.10E-04 | 1.20E-03 | 5.16E-04 | 0.00E+00 | 0.00E+00 | 3.20E-05 | 1.96E-03 |
| 8 | *HNF4A* | 4.02E-05 | 6.02E-04 | 2.89E-05 | 7.33E-04 | 2.42E-05 | 3.74E-04 | 1.80E-03 |
| 9 | *HMOX1* | 1.47E-04 | 5.53E-05 | 3.04E-04 | 4.63E-05 | 3.48E-05 | 1.18E-03 | 1.77E-03 |
| 10 | *FAS* | 1.87E-04 | 2.52E-04 | 6.25E-04 | 1.70E-04 | 2.69E-04 | 3.20E-05 | 1.53E-03 |
| 11 | *APP* | 3.17E-04 | 4.73E-04 | 1.39E-04 | 1.17E-04 | 1.97E-04 | 9.77E-05 | 1.34E-03 |
| 12 | *CYP17A1* | 2.99E-05 | 4.50E-05 | 2.06E-04 | 2.97E-05 | 4.16E-05 | 8.76E-04 | 1.23E-03 |
| 13 | *IGF1* | 3.50E-05 | 4.96E-04 | 6.35E-05 | 5.54E-05 | 3.60E-04 | 1.53E-05 | 1.03E-03 |
| 14 | *PTGS2* | 1.40E-04 | 2.16E-04 | 2.90E-04 | 2.70E-05 | 2.05E-04 | 1.39E-04 | 1.02E-03 |
| 15 | *SOD1* | 2.89E-04 | 2.55E-05 | 2.22E-04 | 2.10E-04 | 2.70E-05 | 2.07E-04 | 9.80E-04 |
| 16 | *BDNF* | 1.97E-05 | 5.17E-05 | 6.01E-04 | 2.24E-05 | 1.01E-04 | 5.04E-05 | 8.46E-04 |
| 17 | *NOS2* | 3.27E-05 | 5.20E-05 | 4.59E-04 | 2.52E-05 | 1.45E-04 | 1.21E-04 | 8.34E-04 |
| 18 | *TGM2* | 2.12E-05 | 9.00E-05 | 3.31E-05 | 7.09E-06 | 3.18E-05 | 5.03E-04 | 6.86E-04 |
| 19 | *GCH1* | 1.48E-04 | 4.13E-05 | 9.22E-06 | 2.58E-04 | 4.14E-06 | 2.06E-04 | 6.66E-04 |
| 20 | *UCHL1* | 1.29E-04 | 7.90E-07 | 6.00E-07 | 5.28E-04 | 9.60E-07 | 1.30E-07 | 6.60E-04 |
| 21 | *IL1B* | 2.90E-05 | 5.13E-05 | 2.85E-05 | 2.10E-05 | 5.10E-04 | 7.69E-06 | 6.48E-04 |
| 22 | *HNF1A* | 1.88E-05 | 1.35E-04 | 1.69E-05 | 2.00E-04 | 3.02E-05 | 1.81E-04 | 5.82E-04 |
| 23 | *APOE* | 5.10E-05 | 1.28E-04 | 6.59E-05 | 4.33E-05 | 2.45E-04 | 1.91E-05 | 5.52E-04 |
| 24 | *IGF2* | 2.88E-05 | 2.32E-04 | 2.61E-05 | 1.65E-04 | 6.39E-05 | 1.35E-05 | 5.29E-04 |
| 25 | *CYP1A1* | 1.42E-05 | 1.04E-05 | 3.93E-05 | 1.18E-05 | 5.55E-06 | 4.47E-04 | 5.28E-04 |
| 26 | *PPARG* | 7.15E-05 | 2.07E-04 | 1.79E-05 | 1.30E-04 | 7.45E-05 | 1.56E-05 | 5.16E-04 |
| 27 | *SLC18A2* | 4.16E-04 | 4.01E-05 | 2.72E-05 | 8.78E-06 | 5.75E-06 | 6.66E-06 | 5.05E-04 |
| 28 | *CD14* | 2.29E-04 | 3.94E-05 | 1.91E-05 | 1.14E-05 | 1.96E-04 | 6.13E-06 | 5.01E-04 |
| 29 | *PINK1* | 9.85E-05 | 1.25E-04 | 8.76E-05 | 5.50E-05 | 8.71E-05 | 2.01E-06 | 4.55E-04 |
| 30 | *INS* | 8.21E-05 | 0.00E+00 | 7.97E-05 | 1.30E-04 | 1.00E-04 | 1.88E-05 | 4.10E-04 |
| 31 | *PARP1* | 1.81E-04 | 7.52E-05 | 3.56E-05 | 2.93E-05 | 5.59E-05 | 1.60E-05 | 3.93E-04 |
| 32 | *NFKB1* | 1.62E-04 | 1.34E-04 | 5.60E-05 | 3.34E-05 | 0.00E+00 | 6.88E-06 | 3.92E-04 |
| 33 | *SLC2A4* | 1.88E-04 | 0.00E+00 | 1.59E-05 | 6.06E-05 | 1.55E-05 | 9.41E-05 | 3.74E-04 |
| 34 | *IDE* | 9.91E-05 | 7.13E-05 | 1.21E-05 | 5.38E-05 | 1.49E-05 | 1.21E-04 | 3.72E-04 |
| 36 | *DRD2* | 1.09E-04 | 4.91E-05 | 6.36E-05 | 1.56E-05 | 1.20E-04 | 8.04E-06 | 3.66E-04 |
| 37 | *GAD2* | 1.79E-05 | 7.99E-06 | 1.68E-05 | 2.67E-04 | 1.21E-05 | 2.78E-05 | 3.50E-04 |
| 38 | *SORBS1* | 3.21E-05 | 0.00E+00 | 1.04E-04 | 1.30E-05 | 1.96E-04 | 4.86E-06 | 3.50E-04 |
| 39 | *CP* | 1.43E-04 | 1.61E-05 | 2.32E-05 | 2.32E-05 | 1.19E-05 | 1.18E-04 | 3.35E-04 |
| 40 | *TH* | 1.64E-04 | 5.27E-06 | 3.49E-06 | 1.32E-04 | 2.75E-06 | 8.15E-06 | 3.16E-04 |
| 41 | *TSC2* | 1.53E-05 | 0.00E+00 | 3.85E-05 | 6.54E-05 | 1.80E-04 | 3.69E-06 | 3.02E-04 |
| 42 | *PON1* | 7.71E-06 | 8.29E-05 | 1.48E-05 | 1.73E-04 | 5.60E-06 | 7.00E-06 | 2.91E-04 |
| 35 | *E2F1* | 3.08E-05 | 1.17E-04 | 1.95E-05 | 3.01E-05 | 8.21E-05 | 5.79E-06 | 2.85E-04 |
| 43 | *CXCR4* | 3.42E-05 | 1.74E-04 | 4.56E-05 | 1.89E-05 | 0.00E+00 | 5.69E-06 | 2.78E-04 |
| 44 | *CDKN2A* | 4.95E-05 | 8.19E-05 | 3.34E-05 | 2.77E-05 | 5.81E-05 | 7.88E-06 | 2.59E-04 |
| 45 | *KCNJ2* | 3.68E-05 | 9.63E-06 | 1.80E-04 | 4.50E-06 | 7.34E-06 | 8.90E-07 | 2.39E-04 |
| 46 | *PPARGC1A* | 8.59E-05 | 0.00E+00 | 1.60E-05 | 9.91E-05 | 1.93E-05 | 1.05E-05 | 2.31E-04 |
| 47 | *HGF* | 1.67E-05 | 4.80E-05 | 2.10E-05 | 1.04E-05 | 8.99E-05 | 8.56E-06 | 1.95E-04 |
| 48 | *OPRM1* | 6.93E-05 | 1.64E-05 | 3.46E-05 | 3.93E-06 | 6.48E-05 | 5.90E-07 | 1.90E-04 |
| 49 | *TF* | 4.58E-05 | 3.04E-05 | 2.36E-05 | 2.87E-05 | 2.40E-05 | 2.41E-05 | 1.77E-04 |
| 50 | *ACE* | 2.33E-05 | 2.06E-05 | 2.16E-05 | 3.08E-05 | 6.03E-05 | 1.68E-05 | 1.73E-04 |
| 51 | *CADM1* | 8.86E-06 | 4.13E-05 | 8.41E-05 | 4.55E-06 | 3.34E-05 | 1.18E-06 | 1.73E-04 |
| 52 | *NQO1* | 2.52E-05 | 4.81E-06 | 8.78E-06 | 5.38E-05 | 1.76E-06 | 3.06E-05 | 1.25E-04 |
| 53 | *GAD1* | 1.45E-05 | 5.59E-06 | 1.94E-05 | 4.08E-05 | 1.15E-06 | 4.07E-05 | 1.22E-04 |
| 54 | *GH1* | 4.26E-06 | 6.78E-05 | 6.50E-06 | 1.13E-05 | 2.16E-05 | 3.76E-06 | 1.15E-04 |
| 55 | *HFE* | 6.71E-05 | 7.37E-06 | 6.62E-06 | 1.02E-05 | 8.94E-06 | 1.20E-05 | 1.12E-04 |
| 56 | *CXCL12* | 1.68E-05 | 4.53E-05 | 2.75E-05 | 1.38E-05 | 0.00E+00 | 5.87E-06 | 1.09E-04 |
| 57 | *ABCB1* | 2.46E-05 | 1.12E-05 | 8.73E-06 | 9.51E-06 | 4.93E-06 | 3.38E-05 | 9.27E-05 |
| 58 | *MAOB* | 2.62E-05 | 4.99E-06 | 6.56E-06 | 1.26E-05 | 2.86E-06 | 3.83E-05 | 9.15E-05 |
| 59 | *BTG1* | 4.42E-06 | 2.90E-05 | 8.75E-06 | 3.89E-06 | 3.93E-05 | 1.25E-06 | 8.66E-05 |
| 60 | *ABCC8* | 4.46E-06 | 2.51E-05 | 3.57E-05 | 9.80E-06 | 2.23E-06 | 8.63E-06 | 8.58E-05 |
| 61 | *PDX1* | 6.06E-06 | 3.74E-05 | 8.36E-06 | 1.61E-05 | 9.14E-06 | 4.64E-06 | 8.16E-05 |
| 62 | *ADH1C* | 7.61E-06 | 3.92E-05 | 7.80E-07 | 3.25E-05 | 3.70E-07 | 0.00E+00 | 8.04E-05 |
| 63 | *CCL5* | 1.44E-05 | 3.18E-05 | 1.91E-05 | 8.35E-06 | 0.00E+00 | 2.33E-06 | 7.60E-05 |
| 64 | *TCF7L2* | 1.13E-05 | 2.25E-05 | 9.84E-06 | 8.55E-06 | 1.77E-05 | 3.25E-06 | 7.32E-05 |
| 65 | *ATF6* | 1.62E-05 | 1.71E-05 | 1.00E-05 | 6.65E-06 | 1.39E-05 | 2.80E-06 | 6.66E-05 |
| 66 | *GPX1* | 3.84E-05 | 2.84E-06 | 2.18E-06 | 1.00E-05 | 1.50E-06 | 1.06E-05 | 6.56E-05 |
| 67 | *CCL2* | 1.04E-05 | 2.89E-05 | 1.42E-05 | 7.16E-06 | 0.00E+00 | 2.60E-06 | 6.32E-05 |
| 68 | *VDR* | 8.29E-06 | 1.41E-05 | 6.71E-06 | 8.37E-06 | 8.68E-06 | 1.56E-05 | 6.18E-05 |
| 69 | *MTHFR* | 1.12E-05 | 4.88E-06 | 1.88E-06 | 2.98E-05 | 2.32E-06 | 1.12E-05 | 6.12E-05 |
| 70 | *IL8* | 1.01E-05 | 2.55E-05 | 1.21E-05 | 1.00E-05 | 0.00E+00 | 2.76E-06 | 6.04E-05 |
| 71 | *KIF11* | 1.11E-05 | 1.34E-05 | 1.79E-05 | 7.16E-06 | 9.12E-06 | 1.18E-06 | 5.99E-05 |
| 72 | *MMP16* | 2.99E-06 | 4.76E-06 | 3.01E-06 | 3.24E-06 | 1.65E-05 | 1.51E-06 | 3.20E-05 |
| 73 | *GSTM1* | 1.11E-05 | 1.73E-06 | 1.33E-06 | 3.94E-06 | 5.70E-07 | 1.29E-05 | 3.16E-05 |
| 74 | *HP* | 5.81E-06 | 4.87E-06 | 3.74E-06 | 3.76E-06 | 8.82E-06 | 1.60E-06 | 2.86E-05 |
| 75 | *NPPB* | 8.80E-07 | 2.50E-06 | 6.81E-06 | 5.40E-07 | 1.64E-05 | 4.70E-07 | 2.76E-05 |
| 76 | *SEMA6A* | 5.00E-07 | 3.89E-06 | 2.06E-06 | 3.60E-07 | 4.98E-06 | 9.00E-08 | 1.19E-05 |
| 77 | *NCAM2* | 1.28E-06 | 2.10E-06 | 2.95E-06 | 3.50E-07 | 3.00E-06 | 1.30E-07 | 9.81E-06 |
| 78 | *SERPINB1* | 2.33E-06 | 1.53E-06 | 6.10E-07 | 1.59E-06 | 2.97E-06 | 5.20E-07 | 9.55E-06 |
| 79 | *NAT2* | 1.29E-06 | 2.90E-07 | 1.20E-07 | 2.30E-06 | 4.00E-08 | 1.46E-06 | 5.50E-06 |
| 80 | *MBNL1* | 4.50E-07 | 1.56E-06 | 8.20E-07 | 7.00E-07 | 6.30E-07 | 1.10E-07 | 4.27E-06 |
| 81 | *PCDH18* | 1.80E-07 | 1.51E-06 | 8.60E-07 | 1.10E-07 | 1.46E-06 | 4.00E-08 | 4.16E-06 |
| 82 | *OLFM4* | 8.00E-08 | 0.00E+00 | 1.80E-06 | 8.00E-08 | 8.00E-08 | 1.00E-08 | 2.05E-06 |
| 83 | *RBMS3* | 1.20E-07 | 2.50E-07 | 1.10E-07 | 1.37E-06 | 6.00E-08 | 6.00E-08 | 1.97E-06 |
| 84 | *TBC1D22A* | 2.30E-07 | 1.40E-07 | 4.10E-07 | 2.30E-07 | 5.00E-08 | 6.00E-08 | 1.12E-06 |
